# Remarks on Dr. Stone's Treatise

**Published:** 1806-11

**Authors:** 


### *To the Editors of the Medical and Physical Journal.*

GENTLEMEN,

HAVING been much surprised in the perusal of Dr. Stone's Treatise on the Diseases of the Stomach, at some of the opinions therein advanced, I take the liberty, through the medium of your Journal, to offer a few remarks upon them; demanded, I think, by the importance of the subject.

I am, &c.

N. S.

Member of the Royal College of Surgeons.

---

To canvas the opinions of medical men merely for the sake of disputation, is beneath the dignity of the profession; but when the express object of a work is to abolish facts which the test of ages has approved, it then becomes a duty to question how far such authority should be the standard of practice, as the welfare of our fellow creatures is involved in the result.

My principal attention will be directed to the practical part of this work, particularly that which treats of emetics. I shall not attempt to follow the author through his anatomical and physiological descriptions, but cannot, however, avoid noticing much useless refinement, with a wish to establish unnecessary distinctions. As one example of many, I would bring his objecting to the terms "peritoneal, muscular, and villous, as coats of the intestine. The name of coat, in anatomy, (he says) should always signify a containing membrane;" but this definition appears to be far from perfect; for that which covers any thing is surely sufficient to constitute it a coat; and will any one be so absurd as to declare, that the tunica vaginalis testis is no coat of the testicle: neither the pleura of the lungs; or that the muscular and elastic coverings of arteries are no coats of the same? Supposing the villous membrane to be the only proper intestinal coat, still the intestine would be unable to perform its functions in propelling the food, &c. without the aid of the muscular coat; hence the uselessness of this distinction.

In

In page 11, the Doctor endeavours to prove, that digestion takes place in the fœtus in utero; the evidences adduced are very equivocal; the office of digestion should seem quite unnecessary to uterine life, when the child needs not food for support, but is wholly nourished by prepared blood from the mother.

All analogical reasoning is much to be distrusted, when applied to explain the action of various substances upon the human body, which is so constituted as to render the effect of external agents extremely uncertain; yet the author builds his theories of the formation of chyle, and of digestion, upon chemical experiments on dead matter, making chylification merely an act of precipitation; "the gastric fluid, a secretion in a mild form, only rendered active by decomposition," though we well know it would dissolve the stomach itself, were it not one of the principles of life to resist the action of this solvent. Allowing the process of solution to be the peculiar office of the gastric fluid, surely no one would thence assert, that either this secretion, or those of the liver and pancreas, however combined, are sufficient to assimilate and animalize discordant matters. Is not this another attribute of the principle of life? It were to be wished the Doctor had made nicer discrimination between the laws which govern animate and inanimate bodies, chemical agency being destroyed by the properties of life.

Respecting the history of diseases I have little to remark; such a formidable assemblage of symptoms mentioned as accompanying simple acidity of the stomach, I believe, is seldom witnessed. In page 49, the author asserts, that the mesenteric glands are not adapted for secretion; whereas, it is well ascertained that the chyle undergoes some change in its passage through them, which could not otherwise happen. Neither can I agree with the Doctor, that the secretion of bile is essential to chylification, because that process goes on when the ductus communis choledochus is obstructed, as frequently is the case in jaundice; or that "the most common kind of marasmus depends upon too much eating," when I believe, that its most frequent origin is in diseased mesenteric glands.

On the subject of poisons there is much room for comment; the Doctor begins by giving us an analysis of some pills containing a considerable quantity of the oxid of antimony, the object of which it is difficult to comprehend,

 unless unless it be to reprobate the liberal employment of this mineral, "the too free use of which leaves a peculiar whiteness of the tongue, resembling a covering of thin paper." This is an effect never observed by Dr. George Fordyce, who, perhaps, used more antimony than any physician in England.

The remark on hyoscyamus being useful in continued delirium, when opium disagrees, I think valuable.

The author next proceeds to give his opinion of *digitalis,* in which he appears to be under the full influence of prejudice. That an incautious use of this plant will produce mischievous effects is indisputable; but is it on that account to be rejected? Do we possess any single article in the Materia Medica which has such influence over the actions of the heart and arteries, and absorbent system, that so much relieves various hydropic complaints, that possesses so much power over the various species of mental derangement?

"It unquestionably appears from *reasoning,* (the author says) that a medicine of which the direct effect is to lessen the muscular action of the arterial system is contraindicated in dropsy." Now, all reasoning in medicine should yield to facts; our inability to explain the action of any particular remedy is no argument whatever against its use; therefore, if repeated experiments have confirmed its utility, (of which there remains no doubt) employed it will be by every unprejudiced practitioner. Again, it is stated, "that few who have taken *digitalis* freely have survived a twelvemonth," a conclusion which cannot easily be admitted. I have been in the habit of employing this substance for many years without ever observing any permanent bad effects; and there are physicians who have most extensively used it, unattended with such consequences. "As to the altered state in which it leaves the stomach," I believe there is no well attested instance on record justifying such a supposition; indeed this would be the only example of vegetable poison leaving such an effect upon the stomach.

I shall pass over the remaining chapters on diseases of the stomach, and proceed to notice the plan Doctor S. recommends for their treatment. Here the first thing which excites my astonishment is, to find the author disclaiming against the use of emetics, when, I believe, it will be readily acknowledged by the most sagacious and experienced, that their early and frequent employment at the the commencement of most of the acute diseases is one of the greatest improvements in modern practice.

The author begins by saying, "that the very common and capricious use of emetics has proved a most pernicious error." I am sorry that the Doctor has made use of the word capricious; for no one, I think, would wantonly employ so active an instrument without having formed some notions of the exact nature of the disease in which he is about to use it, or without having witnessed decided benefit under similar circumstances.

As Doctor S. is so extremely anxious to instruct his poor ignorant brethren in this most important point of practice, it behoves us to trace how far his statement corresponds with what actually takes place in disease, more particularly, as some of the "less informed" may, from experience, be inclined to employ emetics in those diseases in which the Doctor would highly reprobate their use. The question therefore is, what are the immediate and ultimate effects of emetics, and how far is their exhibition right or wrong in certain diseases? This, however, cannot be decided by man's most ingenious reasoning, but by facts alone, and facts there are, rendered incontrovertible by the accumulated evidence of ages.

Before I enter into this discussion, it is but right to notice two pages reflecting upon a class of men most useful to society, apothecaries, "who," our author says, "are determined to exercise the right of prescription, though incapable of appreciating the moderate action of moderate medicines." Now, independant of the justice or injustice of such an assertion, I believe greater powers of perception, and nicer discrimination, are requisite to distinguish the true nature of diseases at their commencement than at any future period—and who is hourly called to the exercise of such talents, if not the apothecary? It is to be lamented, that the Doctor should thus depreciate one class of men, in order to elevate another. I would ask any unprejudiced man, whether pre-eminence in station ought not to be estimated by pre-eminence in the power of doing good? And, supposing a physician to be weighed in the scale of usefulness against the surgeon, midwife, and apothecary, who would be found to preponderate? It is far from my intention, however, to enumerate here the respective merits of each department of medicine; distinctions are made by art or interest, which do not exist in nature, of which this is an instance; my belief is, that that man is best qualified to understand the animal economy my with all its varied derangements, who unites in his practice the three branches of the profession.

Concerning the immediate effect of an emetic, Doctor S. says, it is "to increase the secretion from the stomach, and from all the neighbouring glands, and to excite contraction in the muscular fibres of the stomach and small intestines."

Differing from the Doctor, I believe, that the first effect of an emetic is to reverse the natural peristaltic motions of the stomach, duodenum, and perhaps jejunum, so as to make their contents be thrown upward, afterwards to occasion greater secretion from the glands of the stomach and upper part of the intestinal canal, with subsequent expulsion of the secreted matters: these should appear to be the primary effects of an emetic; but, in natural vomiting, which frequently occurs at the beginning of acute diseases, the case is quite otherwise; for it very generally happens that the whole contents of the stomach are not ejected; for if an emetic be given after a patient has been thus vomiting for a considerable time, a vast quantity of undigested matter will be expelled. I would also maintain, that the taking of warm water infusions of bitter herbs, &c. are insufficient to make the stomach throw off the whole of its contents; this I regard as a fact essential to remember in practice.

Mere evacuation of the stomach, and consequent secretion, are far from being the principal actions of emetics; there are secondary effects of the greatest consequence that demand diligent attention, upon which, however, the author has been totally silent.

After administering an emetic, the person is in the first place very uneasy and nauseated, and at this time the whole of the vessels on the surface of the body are contracted, and the strength universally depressed; vomiting ensues, and general agitation of the whole frame; after which, universal relaxation arises all over the system; then succeed moisture and softness of the skin, with an increase of all the secretions; the same happens in the second and third fits of vomiting, but if we urge it farther by large draughts of water, these specific effects are destroyed.

With the view of breaking the assemblage of symptoms, which takes place upon the attack of disease, it is not indifferent whether we employ a substance that occasions vomiting by simply stimulating, or one that has a specific stimulus for the stomach; if we give too large a dose of most most medicines, vomiting will be the consequence, and the specific power (if it had any) will be destroyed. For example, too large a dose of bark will induce vomiting, not diminish the irritability of the body; antimony, purging, vomiting, or profuse perspiration, not relaxation of the exhalents. Inattention to this circumstance has caused, and is daily causing, serious mischiefs in practice. It is also of primary consequence, that the dose of the emetic be not too large, for then we make it act as a simple stimulant, and every other effect is entirely lost by such administration.

To insure that succession of actions, which has been stated as consequent upon giving such an emetic, as has a peculiar stimulus for the stomach, it is best to employ ipecacuanha and antimon. tartarisat. in combination; the former more certainly to evacuate the contents of the stomach; the latter to produce a more universal shook to the system, as well as to relax the extreme vessels, which effects are more certain if exhibited in bed, when the body is less subjected to irregularities of circulation.

"It appears further, (says the author) that the attachment of the villous membrane is injured by the contractions of the stomach when violent and complete;" but experience only tends to invalidate this opinion; we are indeed told, "that nothing is more important than to avoid making facts bend to theories;" and yet, perhaps, a more complete illustration of this error cannot be found than in the work before us, where the act of vomiting is almost invariably held up as dangerous; now, I cannot hesitate to assert, that there never was a more mistaken idea, or one more fraught with mischievous consequences than that vomiting being an unnatural, is a dangerous process.

Two fallacies, the Doctor says, have taken place as to the supposed benefit of emetics: "one when too much or improper food has been taken, a spontaneous vomiting coming on, often relieves the patient." Now, I have stated, that most frequently spontaneous vomiting does not evacuate the contents of the stomach, and "as for a mild purgative answering the purpose better," I must beg leave to differ; for its action is totally different, and quite incompetent to effect those changes in the system that have been mentioned as the result of employing an emetic power. "The other fallacy is, the belief of practitioners that great good has been done, because much bile is often thrown up;" this may be, and frequently is, the language of nurses and old women, but certainly not of medical men, men, who cannot be ignorant that bile is very rarely found in the stomach; that when present, it is the consequence of repeated acts of vomiting. What test the Doctor can have to prove that the quantity of bile occasionally evacuated by this ignorance of practitioners, "has been more than the gall bladder and biliary ducts could possibly contain," cannot be known; one thing is certain, that the smallest portion of bile in the stomach will communicate its colour to whatever may be the contents, which circumstance must destroy any thing like accurate analysis.

The Doctor next observes, that "the particular effects of an emetic, already stated, should be duly considered before we administer them in any disease." I would seriously ask the author, whether our very best remedies have not their attendant inconveniencies; whether medical men are not continually placed in such circumstances that they must submit to some evil, sagacity being exercised in choosing the least. Granting then that some temporary disadvantages may now and then result from the use of emetics, are they ever proportionate to the benefit received, or for a moment to be put in competition with the life of the patient?

In another place, the author strongly objects to the use of antimony as an emetic to children; here again astonishment would be excited, were we not fully aware of the Doctor's irresistible prejudice to this remedy: He grounds his argument upon "having known more than one child killed by its use;" but will he assert that, because two or three times in cynanche maligna, in scarlatina, and in measles, the application of a blister to the throat or chest has been followed by mortification; we ought thence to be deterred from ever employing the same remedy in future. The Doctor here builds his evidence on one or two solitary (perhaps singular) cases, regardless, or rather unwilling, to acknowledge the hundreds of persons to which antimony, in one form or another, is daily administered by mothers as well as medical men, not only without inducing bad effect, but with decided benefit. That a valuable remedy may have its defects cannot be doubted; few will deny that this has one, or perhaps more; but is it on that account to be discarded? Is antimony to be abanded because now and then it causes violent contractions of the stomach, or partial evacuations? Rather than such unqualified disapprobation, how much more useful to the the reader, if the Doctor had been so kind as to give us the history of the action of antimony upon the living body.

Before I notice the effects of emetics in particular diseases, I would observe, that whatever occasions a violent perturbation, or universal shock to the constitution, more especially at the commencement of disease, has a wonderful influence in cutting short or in very much moderating the symptoms of the disease; which effect would seem to depend on the series of symptoms being disturbed or broken.

It is on this principle, I conceive, that Currie's plan of affusion of cold water has been so generally serviceable in fevers, &c. but nothing should appear to possess this power in a much greater degree than emetics. Another reason for their general employment at the commencement of disease is to excite re-action and consequent relaxation of the extreme vessels; another is to arrest, alter, or destroy the morbid actions which are going on in the secretory vessels in many acute diseases; still one more, not less important, is to dispose the body to be acted upon by agents, which, before their application, would have had little or no influence, that is to say, to render the body susceptible of the specific action of individual remedies, be the disease what it may.

I shall now mention the particular diseases in which emetics have the author's sanction or disapprobation: the first case where he strongly recommends an emetic, "is in the state resembling apoplexy, arising from fulness of the stomach;" here I would deem it a very equivocal remedy; nay, if I can believe the testimony of some, it has evidently proved mischievous. But among the extraordinary things we meet with in this Treatise, the Doctor reprobates the employment of emetics in fevers, where nausea is no unfrequent symptom at the commencement; not depending, however, as he asserts, "on febrile irritation, or sympathy with the head," but actually on the presence of viscid and indigestible matters; for no sooner (generally speaking) is an emetic exhibited but the head is relieved; if we were to wait agreeably to the Doctor's instructions, "until it be ascertained that the contents of the stomach cannot be corrected or carried off by the more mild operation of a gentle purgative," we should lose the very best opportunity of doing good. So extraordinary are the author's opinions, that it is not harsh to suppose the one or the other of two things, either that he has not had much experience in these diseases, or that he is determined determined to support his hypothesis in opposition to the most unequivocal testimonies.

If we possess one remedy more powerful than another, either in cutting fevers short, or in rendering them more mild in their progress, I would ask the candid and unbiassed practitioner, whether that remedy be not an emetic?

The author next proceeds to scarlatina and cynanche maligna, in both of which he again decries their use; he also adds the reason why the practice has so generally obtained, "namely, to evacuate by the mouth the offensive matter which is found lodged in the fauces, and thereby to prevent the mischievous effects of large quantities of this matter when passed into the stomach." I believe that this is not the principle on which emetics are employed by most practitioners; that the ulcers are the effects of an erysipelatous inflammation of the mucous membrane of the throat, that all the occasionally attendant symptoms of scarlet efflorescence of the skin, head-ache, nausea, heat of skin, extreme lassitude, &c. arise out of this peculiar inflammation, and will subside upon its removal. Shortly afterwards the author asserts, that by the use of a contra-yerva gargle, formerly prescribed by Dr. Fothergill in this complaint, he has cured cynanche maligna; "that by it the stomachs of patients have been made to bear diet, &c. and have rapidly recovered." Unless it were known that this disorder had a natural cure of its own, I should be surprised at such a relation. In opposition to the Doctor's experience, I must strenuously urge the importance of an emetic at the commencement of this disease, or even at the fifth or sixth day, as by it I have repeatedly had the satisfaction of seeing the disease completely cut short, or its violence greatly moderated; invariably it has disposed the stomach to receive the impression of remedies, which before it would have resisted; nay, I feel convinced, that if there be a disease, in which their repetition is justifiable, it is in this, having witnessed most decided benefit from repeating such a remedy at various periods of the disease.

The use of emetics, we are again told, is only sometimes admissible in croup; but I am compelled to declare, that abundant experience authorises their universal employment at the commencement of this disease. We know of no remedy, if used early, that so certainly destroys the morbid actions which are going on in the trachea, eventually forming that adventitious membrane which destroys the patient.

In

In hooping cough, we learn, the occasional use of emetics is beneficial; in this I coincide with the Doctor.

He very justly reprobates their use in *all* inflammatory affections of the thorax, but there are many affections of the lungs in which the effects of vomiting are manifestly useful; not however, I presume, "simply from the pressure of the contents of the stomach being removed," but from the relaxation of the exhalents of the lungs, and consequent separation of whatever may be contained in the extreme branches of the bronchiæ; it is on this principle, I conceive, that the use of emetics in asthma and consumption do occasionally afford much relief.

That the same effects will ensue, "much more safely and easily by the operation of a purgative," I greatly doubt; indeed, from much patient observation, I am led to believe that purgatives rather do harm in many affections of the chest. Be that as it may, secretion from the exhalents of the lungs is not equally increased by the action of a purgative as by an emetic.

Who would think of employing a purgative to relax the cuticular vessels? Indeed, if they were already in a state of relaxation, it would be altered, or cease altogether upon the action of the purgative.

"It is reasonable to suppose, (says the author) that hæmorrhagy from the lungs in phthisis will be the effect of emetics; and that though the efforts to vomit relieve the lungs at the instant, yet that is to be expected which in fact takes place, the shock given to the lungs increases the suppurative disposition in them, and the load of matter upon them is quickly increased:" the validity of this opinion may justly be called in question. Reasoning à priori one might expect mischief to accrue; but this is not allowable in medicine, where facts, however inexplicable, should form the basis of practise, and facts we have, contradicting such a supposition. I verily believe, indeed, that emetics often induce a diminished action of the heart and arteries, and that even during their operation. Uterine hæmorrhage is suspended by vomiting; there are also instances of persons labouring under hæmoptysis, who were continually sick during a long sea voyage, without experiencing the least increase of hæmorrhagy, vide Heberden's Commentaries, and Dr. B. Robinson's Works. That emetics or any other remedy should not cure hæmoptysis is not wonderful, depending, as it does most frequently, upon a diseased state of the coats of the vessels.

"In

"In epilepsy, and chorea sancti viti, the author thinks emetics sometimes useful, though in the former disease (he says) they are likely to be injurious from the sudden propulsion of blood to the head; in fact, there are instances when their administration have aggravated the symptoms." A few lines afterwards he states, "that some absorption probably takes place from the brain whenever emetics have been successfully given." Absorption of what, I would ask? It were to be wished the Doctor would give us more definite ideas of the true nature of epilepsy; for one instant we are led to believe that it consists in, or is intimately connected with, fulness of the vessels of the head; the next, that there is an effusion of something which is to be absorbed; the fallacies of which opinions need not a moment's controversy.

After some general observations on dropsy, the author says, "It should never be forgotten, that every emetic gives a violent shock to the constitution as well as to the stomach," and closes the chapter with this strange remark: "It is to be hoped the time will soon come when these medicines may never be exhibited *except in cases of poison or immediate suffocation*, without the sanction of some physician of sagacity and experience." Contrary, however, to such an opinion, pregnant with mischief, I hesitate not to affirm, and all times and experience will render the assertion fully vindicable, that emetics are eminently useful in the following diseases.

1st, At the commencement of all the varieties of the common continued fever of this country.

2dly, Upon the attack of all remittent and intermittent fevers, the effects of which are to render the intermissions more perfect, and to dispose the body to be acted upon by the necessary remedies.

3dly, In all the gradations of cynanche maligna and scarlatina.

4thly, In erysipelas, to dispose the stomach to receive the necessary quantity of bark.

5thly, In epileptic and hysteric spasms.

6thly, In convulsions, to take off irregular actions.

7thly, In dysentery, to throw the circulation upon the surface of the body.

8thly, In croup.

9thly, In various species of impeded respiration.

10thly, In all the species of insanity, from the slightest aberration of intellect to complete mania furibunda.

11thly, In the beginning of acute rheumatism.

12thly,

12thly, To increase secretion from the liver and neighbouring parts.

The limits of this paper will not admit me to follow the author through the remaining chapters of his Treatise, which really appear to contain nothing very interesting. In chapter IV. he strongly recommends powerful emetics when the patient is in an apoplectic state from repletion; this is rather strange doctrine. In some of the most valuable remedies of the materia medica, the Doctor should seem to have been peculiarly unfortunate in his trials, Ol. Ricini is another instance; that its purgative quality should vary much cannot be doubted by any one at all acquainted with the preparation of this drug, but that it should reproduce spasm in colica pictonum, and bloody flux in dysentery, are effects to which the experience of few will bear testimony.

As the Doctor is so extremely tenacious of preserving distinctions between medical men, I would advise him to have kept to his own particular department: No surgeon, we apprehend, would think of curing a node (which is a venereal inflammation of the substance of a bone) by mercurial frictions upon the surface of that bone; upon the skin, for example, when situated in the tibia, as recommended by the Doctor.

I am sorry to observe many succeeding pages occupied in useless refinement, as to the method of affecting the liver by mercury. In schirrosities of the liver, consequent upon hard drinking, the author enjoins total and immediate abstinence from all spirit as the best mode of treatment; this, however, is very questionable; few, I believe, will readily adopt such a practice, for we are so constituted, that abruptly and suddenly to take off an accustomed and violent stimulus cannot be practised with impunity.

What strikes one as most singular in the miscellaneous observations is, to find the author recommending purgative medicines in the early stages of contagious fever; at the same time strongly reprobating their use in scarlatina. Assuredly, we have no more command over the action of purgatives in typhus than in scarlatina, and they so frequently produce profuse evacuation, as to render them at best ambiguous remedies. So far from having discovered "that the patients have with greater certainty recovered, and that the time of duration of the fever has been shortened by purgatives," my experience has induced a contrary belief, that partial evacuations are much more likely to to happen in the course of the disease; in fact, that fever is thus rendered irregular, and would have finished its various stages much more safely without the interposition of art.

In whatever light this Treatise is viewed, whether as destined for the younger practitioner (which some have supposed) or for those more conversant with disease, its general evil tendency is but too conspicuous; for the author has taken uncommon pains to depreciate one of the most valuable remedies we possess, by a judicious administration of which we are frequently enabled suddenly to arrest the progress, and to mitigate the violence of a class of diseases which probably destroys two-thirds of the human race.

